# Controlling the spread of Hiv among long haulage workers in Nigeria

**DOI:** 10.1186/1742-4690-9-S1-P107

**Published:** 2012-05-25

**Authors:** Evans Benjamin, Taiwo Kelvin Igie, Monday Udoh

**Affiliations:** 1 Foundation Aid Solution for Talent Empowerment and Development, Ikeja Lagos, Nigeria

## Introduction

The study investigated the effect of the transport industry on the transmission and spread of HIV/AIDS. Transport workers are highly mobile and spend long intervals away from the comforts of their homes. They are often involved in risky sexual behaviours that make them vulnerable to HIV infection, and so constitute carriers in the spread of the pandemic.

## Method

The study entailed interviewing more than 1,000 long haul drivers and workers with the objective of inducing frank talk to assess their sexual habits enroute their long hauls. The limitation of this study was their insistence on anonymity to avoid adverse effects on their social status and marriage stability.

## Result

More than 80% of interviewees had more than 20 female friends stationed at the villages on highways across the country. 60% of the promiscuous group knew about condoms but never used them. To them, what was the use doing it if you could not have the real thing. Sadly, some of the interviewees stated they had no other pleasurable indulgences in life other than sex, and if they were to die doing the only thing they enjoyed then who is complaining.

## Conclusion

The NURTW was advised:

Create rest stations along the nation’s highways, with lodging, canteen, games, TV/Video sets, and other recreational facilities for drivers and motor boys.

Provide GSM phone facilities for workers on long distance engagements to allow them keep in touch with their families.

Organize seminars to educate workers on the implications, prevalence and management of HIV/AIDS.

Provide medical test and care facilities at those rest stations for the quiet testing of workers for HIV/AIDS and dispensing of necessary drugs to sufferers.

